# Supplementary material for: Engineering Polypropylene–Calcium Sulfate (Anhydrite II) Composites: The Key Role of Zinc Ionomers via Reactive Extrusion
Source: Polymers (Basel). 2023 Feb 5;15(4):799. doi: 10.3390/polym15040799 (PMC9959175; doi:10.3390/polym15040799)
Supplement: Supplementary file 1 [file polymers-15-00799-s001.zip › polymers-2189908-supplementary.pdf]

For reasons of clarity, the codes and compositions of PP samples concerned by the Supplementary Material are mentioned below:

Codes and compositions of PP samples produced with internal mixers

**Sample code**	**PP, wt.%**	**AII, wt.%**	**Modifier, wt.%**
PP	100	-	-
PP-ZnDA	98	-	2
PP-20AII-PPMA1	75	20	5
PP-40AII	60	40	-
PP-40AII-PPMA1	55	40	5
PP-40AII-ZnDA	58	40	2

Codes and compositions of PP samples produced with twin-screw extruders (TSE)

**Sample code**	**PP, wt.%**	**AII, wt.%**	**Modifier, wt.%**
PP (TSE)	100	-	-
PP-20AII-ZnDA (TSE)	78	20	2
PP-40AII-ZnDA (TSE)	58	40	2
PP-40AII-PPMA2 (TSE)	56	40	4

Abbreviations: AII: $CaSO_4$ β-anhydrite II; ZnDA: Zinc diacrylate; PPMA1 and PPMA2: PP modified with different percentages of maleic anhydride (details in the section Materials of the manuscript).

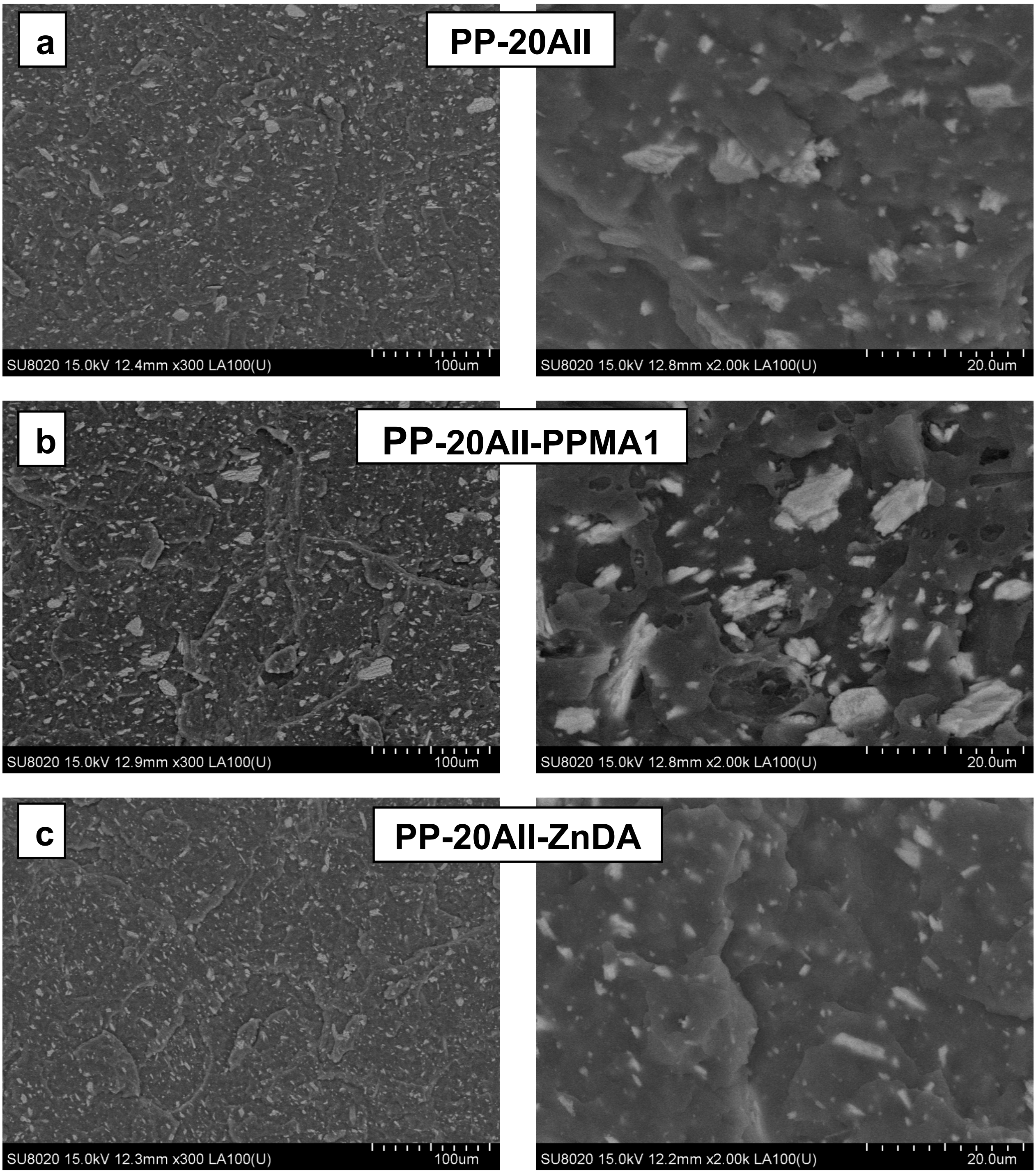

Figure S1. (a–c). Selected SEM pictures (LA-BSE) at different magnifications on the cryo-fractured surfaces of PP composites filled with 20% AII: (a) PP-20AII, (b) PP–20AII–PPMA1 and (c) PP–20AII–ZnDA.

Short comments: *The PP composites filled with 20% AII (concerned by Fig. S1) are obtained using internal mixers. Well-distributed/dispersed particles are evidenced by the SEM micrographs performed at high magnification, especially for the PP-20AII-ZnDA and PP-20AII samples. The PP–20AII–PPMA1 shows somewhat different morphology. It is worth mentioning that the large aggregates are not observed, whereas such quality of distributive mixing within the hydrophobic PP matrix is obtained without any previous surface treatment of the hydrophilic filler.*

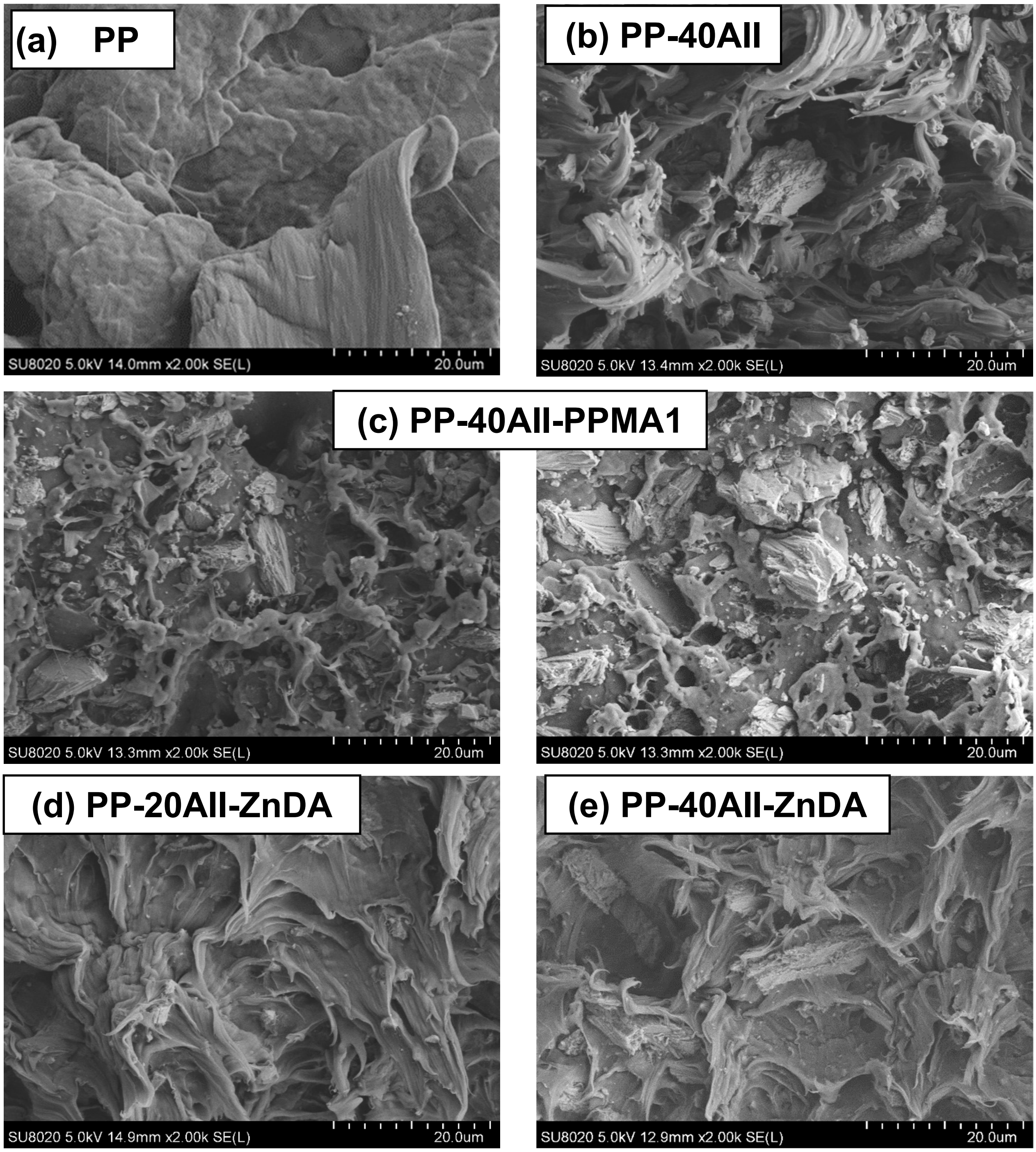

Figure S2 (a-e). Representative SEM images (SE mode) of PP and PP-AII composites performed on the fractured surfaces obtained by tensile testing: (a) PP; (b) PP-40AII; (c) PP-40AII-PPMA1, (d) PP-20AII-ZnDA; (e) PP-40AII-ZnDA.

Short comments: *By comparing to the other samples, the SEM micrographs of PP-AII-ZnDA composites suggest a stronger interfacial adhesion between the PP matrix and filler (better wetting, numerous regions of contact between microparticles and PP matrix, the particles are more deeply lodged within the polymeric matrix, etc.), which explain their good tensile strength. On the other hand, the debonding and the pull-out of particles, is more specifically seen for the other composites.*

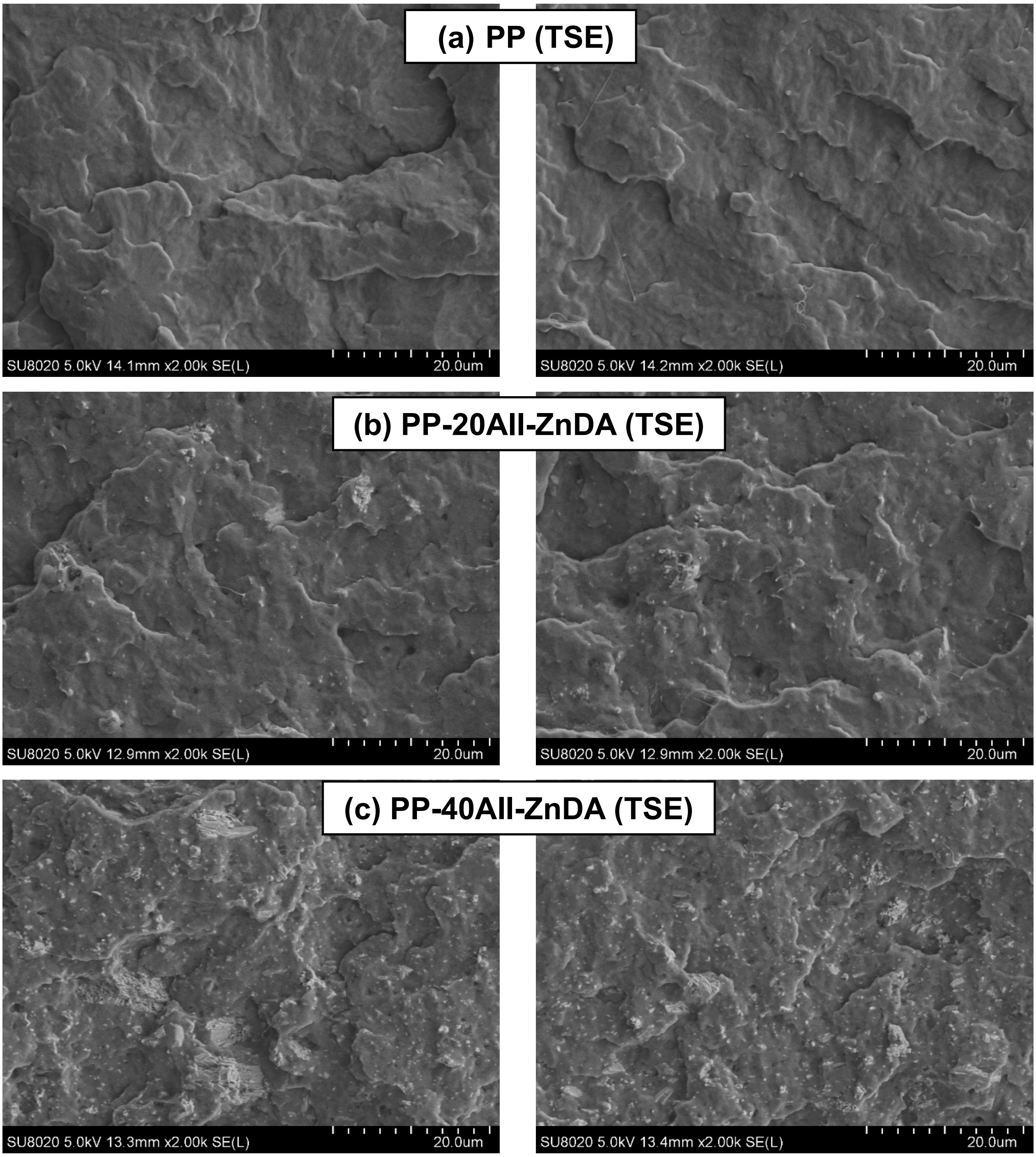

Figure S3 (a-c). SEM images (SE mode) of PP (TSE) and PP–AII-ZnDA (TSE) composites performed on the fractured surfaces obtained by impact testing at room temperature.

Short comments: *The SEM micrographs (Figure S2b and S2c) of the fractured surfaces obtained by impact testing at high energy/deformation rate (respectively, 3.9 J and 3.46 m/s) suggest that the filler has quite good adhesion to the PP matrix, as the pull-out of particles from the matrix, or important signs of debonding at the interface polymer-fille, are not observed. Besides, the distribution/dispersion of AII microparticles and their good wetting/surrounding by the polymer matrix is noticeable.*

Table S1. Comparative properties of neat PP, PP modified with 2% ZnDA, and PP-40% AII composites modified by REx with 4% PPMA2.

**Properties/ samples ⇒**	**PP**	**PP-ZnDA**	**PP-AII-PPMA2**
**Mechanical: tensile properties**			
• Maximum tensile/yield strength, MPa	37 (±1)	34 (±2)	27 (±1)
• Tensile strength at break, MPa	30 (±1)	29 (±2)	25 (±1)
• Young's modulus, MPa	1570 (±70)	1580 (±80)	2150 (±50)
• Nominal strain at max strength, %	7.3 (±0.2)	7.2 (±0.6)	3.0 (±0.3)
• Nominal elongation at break, %	11.1 (±0.7)	11.2 (±0.7)	3.9 (±0.3)
**Izod impact resistance, kJ/m^2**	2.3 (±0.4)	3.1 (±0.4)	1.5 (±0.1)
**Thermal properties (DSC)**			
• Peak of crystallization temperature, °C	116	122	126
• Peak of melting temperature, °C	163	163	164
**Equipment for melt-blending**	Internal mixer	Internal mixer	Twin-screw extruder
**Specimens for mechanical testing produced by**	Compression molding	Compression molding	Injection molding

Short comments:

For additional information and for comparative reasons, the unfilled PP composition modified with 2% ZnDA was prepared using internal mixers and characterized under similar conditions.

By comparing to the neat PP (without any additive), the mechanical properties of PP-ZnDA are remaining almost comparable, except the impact resistance, which has increased from 2.3 kJ/m^2 (neat PP) to 3.1 kJ/m^2 for the reactive modified PP.

As it was mentioned in the manuscript, the realization of PP-AII composites modified/compatibilized by REx with PPMA2 (i.e., PP containing 0.8-1.2% grafted MA) was also considered, but unfortunately, the mechanical properties (i.e., the tensile strength and impact resistance) were less promising than using ZnDA.

NB: PP-40AII-ZnDA (TSE) composites were characterized by a σ_t of 35 MPa and an impact resistance of 2.8 (±0.1) kJ/m^2, more information can be found in the manuscript.
